# Human intestinal models to study interactions between intestine and microbes

**DOI:** 10.1098/rsob.200199

**Published:** 2020-10-21

**Authors:** Arturo Aguilar-Rojas, Jean-Christophe Olivo-Marin, Nancy Guillen

**Affiliations:** 1 Instituto Mexicano del Seguro Social, Unidad de Investigación Médica en Medicina Reproductiva, Unidad Médica de Alta Especialidad en Ginecología y Obstetricia No. 4 ‘Dr. Luis Castelazo Ayala’, Av. Río Magdalena No. 289, Col. Tizapán San Ángel, C.P. 01090 Ciudad de México, México; 2 Institut Pasteur, Unité d'Analyse d'Images Biologiques, 25 Rue du Dr Roux, 75015 Paris, France; 3 Centre National de la Recherche Scientifique, UMR3691, 25 Rue du Dr Roux, 75015 Paris, France; 4 Centre National de la Recherche Scientifique, ERL9195, 25 Rue du Dr Roux, 75015 Paris, France

**Keywords:** intestinal cell culture, organoids, three-dimensional (3D) scaffolds, organ-on-a-chip, microbiota, parasites

## Abstract

Implementations of suitable *in vitro* cell culture systems of the human intestine have been essential tools in the study of the interaction among organs, commensal microbiota, pathogens and parasites. Due to the great complexity exhibited by the intestinal tissue, researchers have been developing *in vitro*/*ex vivo* systems to diminish the gap between conventional cell culture models and the human intestine. These models are able to reproduce different structures and functional aspects of the tissue. In the present review, information is recapitulated on the most used models, such as cell culture, intestinal organoids, scaffold-based three-dimensional models, and organ-on-a-chip and their use in studying the interaction between human intestine and microbes, and their advantages and limitations are also discussed.

## 1. Introduction

The gastrointestinal (GI) tract, is the principal internal barrier of the body that separates human tissues from the external environment, including food, antigens, toxic molecules, xenobiotics and microbes. In the GI barrier, cells must respond to and survive all of these multiple stimuli; they must also ensure the capacity of the intestinal tissue to allow efficient transport of essential nutrients, these facts indicate that the intestinal ecosystem has significant functional complexity. Understanding the complex interaction among all of the elements present in the intestinal environment has been a main objective of research for the comprehension of homeostasis and diseases, including inflammatory bowel disease (IBD), cancer and microbial infections (table 1).

**Table 1.** Overview of intestinal models used to study interactions between the intestine and microbes, and discussed in this review.

model	commensal bacteria	pathogenic bacteria	parasite
tissue explant. Portions of organs maintained in culture by means of specialized media, substrates and atmospheres these models are commonly used to study human cells and parasite interaction, principally in human obligate pathogen advantages: the presences of mucus layer, immunological, neuronal and vascular responses. Models that recapitulate microenvironment observed during human pathologies disadvantages: difficulty in obtaining tissue samples and also, they show short duration of viability. There are genetic variations among the samples, which in some cases affect the interpretation of the data	—study of microbiome. Demonstration of the protective effect of lectin-like protein, ZG16, as protector against Gram-positive bacterial infection [73] —study of probiotic and commensal *Escherichia coli* strains. Demonstration that outer membrane vesicles (OMVs) secreted by *E. coli* Nissle 1917 and *E. coli* ECOR12, is an effective system to communicate human host and bacteria to trigger specific responses [74] —study of commensal bacteria. In ileal explants from Crohn's disease (CD) patients, incubation with commensal bacteria *E. coli* or Lactobacillus *casei* the alterations observed in CD samples were partially recovered [75]	—study of early events of *Shigella flexneri* infection. Interaction between human tissue and the invasive strain of *S. flexneri* induce desquamation and reduction of epithelial height and neuronal alteration of submucosal neurons [76] —evaluation of cholera toxin B subunit (CT-B) from *Vibrio cholerae*. Mucosal explants from CD patients incubated with recombinant CT-B demonstrated the effects of CT-B as an inhibitor of mucosal Th1 cell signalling [81]	—study of virulent factors of *Entamoeba histolytica.* Demonstration of cysteine proteinase A5 activity as a modulator of human metalloproteinase-3 to trigger trophozoite invasion [77]. Transcriptome comparisons between virulent (HM1:IMSS) and non-virulent (Rahman) strains, to provide a global view of the gene expression profile of each strain [78]. In different contexts, was carried out the transcriptome analysis of gene expression of *E. histolytica.* The major transcriptome changes were observed in human explants [79] —study of gene expression of *Cryptosporidium* *parvum* or *Cryptosporidium hominis* during intestinal invasion. During tissue invasion, both strains showed overexpression of osteoprotegerin (OPG) and inhibition in the OPG ligand tumour necrosis factor-related apoptosis-inducing ligand (TRAIL) b via OPG. [80]
organoid. Miniaturized intestine that derives from proliferation of the intestinal stem cells (ISC) and show tissue structures such as villus-like domains with cellular polarization and crypt-like domains with proliferative regions these models are currently used as a platform in pharmacology studies and also as host–bacteria and host–virus interaction models principally, in cases that lack a suitable animal model advantages: their major, strengths are the presence of 3D structures, cellular polarization and presences of several epithelial-cell lineages organoids can be developed from biopsy derivate from healthy samples or from patient samples in two weeks disadvantages: the biggest disadvantage is the need to manipulate the structure to establish the interaction between the organoid and pathogens. At the moment, there are three major methods to stablish host–microbe interaction studies:	—evaluation of microbiota-derived molecules. In organoids deriving from duodenal biopsies from gluten-intolerant patients, the barrier function was improved after the microinjection of microbiota-derived molecules [92] —study of regulatory effects of *Lactobacillus reuteri* in damaged mucosal barrier. By co-culture of mouse intestinal organoids and lamina propria lymphocytes, it was demonstrated that the presence of *L. reuteri* D8 was effective to repair the damaged epithelium caused by TNF-α treatment [93] —evaluation of non-pathogenic strain of *E. coli* in immature human epithelium. In human organoids, it was shown that microinjection of non-pathogenic *E. coli* generated a close host–microbe interaction in naive epithelium. This interaction increases antimicrobial peptide production, maturation of mucus layer, and improve of barrier function [94]	—study of earliest stages of infection of enterohaemorrhagic *E. coli.* Human colonoids, were used to generate a differentiated monolayer in trans-wells inserts. Their infection with enterohaemorrhagic *E. coli* showed high levels of the serine protease EspP. This protein sequentially targets the Mucin-2 and protocadherin-24 to allow the bacterial attachment to the epithelium [95] —infection by *Clostridium difficile.* Microinjection of *C. difficile* showed reduction of MUC2 production, but no changes in mucus oligosaccharide composition [96]. Likewise, a marked epithelial disruption and loss of paracellular barrier promoted by the toxin A were observed [97]. Colonoids derived from Wnt receptor Frizzled 7 (FZD7) KO mice, show that the toxin B is targeted by FZDs receptors in the colonic epithelium [98] —infection with *Salmonella enterica* serovar *Typhimurium.* Microinjection of *S. Typhimurium* induced changes in the human transcriptional pattern and sustained bacterial invasion [100]	—study of the life cycle of *Cryptosporidium* spp. Organoids derived from human small intestine were microinjected with *Cryptosporidium*. This model showed substantial physiological relevance by its ability to complete the complex life cycle of parasite [107,108]
microinjection, mechanical shearing and plating, and by making monolayers	—evaluation of non-pathogenic strain and pathogenic strain of *E. coli*. Microinjection of commensal strains and pathogenic strain O157:H7 of *E. coli* showed rapid growth. The commensal strain did not cause damage suggesting the positive effect of mucus production. Conversely, a loss of actin and epithelial integrity was observed with O157:H7 [99]	—infection with *Helicobacter pylori*. Microinjection of *H. pylori* was used in a model to study bacterial infection [102]. Also, bacterial infection resulted in rapid association of CagA with the c-Met receptor and the induction of epithelial proliferation [101]. Infection induces epithelial proliferation and c-Met phosphorylation [103] —infection with *Shigella flexneri.* Human enteroids were used to generate epithelial layer in trans-well inserts. Their infection showed basolateral invasion and disruption of tight junctions. Finally, bacterial replication, actin tails and increase in proinflammatory signals were observed [104] —infection with pathogenic *E. coli*. Polarized organoids were disaggregated and grown on trans-well inserts. They demonstrated novel adherence phenotypes in various strains of pathogenic *E. coli* [106]	
scaffold-based 3D models. Matrices made with synthetic or natural materials for the deposition of cells to mimic the architecture of the intestine and promote the acquisition of tissue characteristics these models are commonly used to study human cells and bacterial or parasite interactions, principally in human obligate pathogens advantages: their major strengths are the presence of 3D structures, cellular polarization and presence of several cell lineages in some models, the major advantages are their versatility. Components can be varied independently according to the study objectives. There is the possibility of combining 3D scaffolds and cells derived from human intestinal explants or organoids disadvantages: the biggest disadvantage is their lack of physical forces presents in tissues	—study of *Lactobacillus gasseri* and *E. coli* Nissle 1917 as protective agents of the intestinal tract. The therapeutic potential of *L. gasseri* and *E. coli* Nissle 1917 against *Salmonella typhimurium* and *Pseudomonas aeruginosa* was demonstrated [125] —culture of probiotic, *Lactobacillus rhamnosus* GG. Development of bioengineered human intestinal tissues that mimic *in vivo* luminal oxygen levels. This system supports the growth of dominant anaerobic probiotic bacteria, *Lactobacillus rhamnosus* GG [128] —study of commensal bacterial strains. Development of a scaffold-based 3D model that contained enterocytes, goblet and immune-like cells. The cells were exposed to synthetic commensal microbial community and LPS from *E. coli* O111:B4 strain. This interaction promotes the adhesion of specific bacterial strains, *Veillonella parvula*, to stimulate the epithelium barrier function and interleukin production [134]	—infection with *Salmonella typhimurium.* The human intestinal villi epithelium was mimicked in a hydrogel scaffold. Using this model it was possible to establish the importance of MUC17 during bacterial infection [126] —infection with *Yersinia pseudotuberculosis*. The human intestinal lumen was replicated in a 3D porous scaffold. In to evaluate the importance of mucus and the establishment of low oxygen to study bacterial interactions/colonization [128] —infection with *Campylobacter jejuni.* A decellularized extracellular matrix scaffold and reseeded with human Caco-2 cells was designed to study host–pathogen interactions. The infection with *C. jejuni* replicated some pathogenic processes previously observed in animal models and showed new virulent factors involved [132]	*—infection with Cryptosporidium parvum.* It was possible to reproduce the microenvironmental conditions of the intestinal tract to support the life cycle of *C. parvum* in a bioengineered 3D human intestinal tissue model. This system has opened the possibility to evaluate host–parasite interactions and identify new drug targets [129,130] —study of the early steps of *Entamoeba histolytica* infection. The general aspects of the human colon were reproduced in a 3D model, which highlighted the importance of several virulence markers previously observed and also, described new molecules and regulatory factors involved in the amoebic invasive process [136]
organ-on-a-chip. These are micropatterned synthetic surfaces that support the correct spatial arrangement of the cells and help to control gradients of biomolecules by means of microfluidic applications these models are currently used as platform to study host–microbe interaction models for long periods of time. Those models are used principally to study bacterial interaction in cases that lack a suitable animal model advantages: their major advantage is the ability to reproduce the physiology of the human gastrointestinal tract in 3D context, in the presence of flux fluid, peristaltic motion and oxygen gradient disadvantages: their principal weakness lies in the implementation of highly complex structures requiring qualified personnel for their design, and PDMS, the material most commonly used for chip construction, which absorbs small hydrophobic molecules that could interfere with some drug-screening studies	—infection with *Lactobacillus rhamnosus* GG. Organ-on-a-chip support long cultivation of *L. rhamnosus* GG with improved barrier functions and without compromising epithelial viability [142] —iIntestinal interaction among commensal and pathogenic bacteria and intestinal cells. The co-cultivation of commensal microbes and human intestinal epithelial cells suppress villus injury induced by enteroinvasive *E. coli* (O124:NM) [145] —study of complex communities of anaerobic and aerobic commensal bacteria and intestinal epithelium. The control of physiological oxygen gradients was possible in a gut-on-a-chip model, which grew aerobic and anaerobic human microbiota. The model established the importance of oxygen gradient in barrier function and maintaining relevant levels of microbial diversity [149] —study of *Lactobacillus rhamnosus* GG and *Bacteroides caccae* under anaerobic conditions. A microfluidics-based model demonstrated that the co-culture of both bacterial strains promotes the transcriptional response in epithelial cells, which is distinct from that of a co-culture solely comprising *L. rhamnosus* [150]	—infection with *Shigella flexneri*. A gut-on-a-chip model demonstrated the positive impact of peristalsis during the epithelial invasion of *S. flexneri* [144] —infection with enterohaemorrhagic *E. coli*. An organ-on-a-chip model demonstrated that during infection by enterohaemorrhagic *E. coli*, administration of human microbiome metabolites promote the expression of virulent bacterial factors and increased epithelial injury [148]	no information available

### 1.1. Many different cells build the intestine

**Figure 1.**
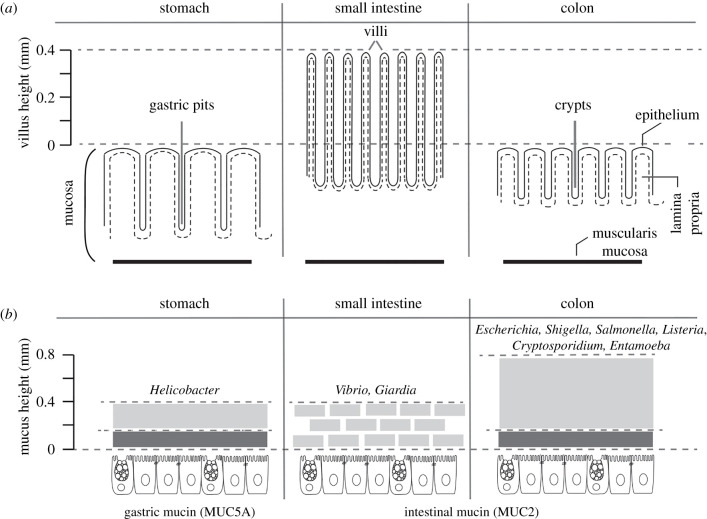
(*a*) Schematic of gastric pits, villi and crypts present in stomach (i), small intestine (ii) and colon (iii), respectively. The relative villus height in the small intestine is shown (millimetres); these structures are absent from the other intestinal compartments. The mucosa layer and its main components, such as the epithelium (continuous and dashes lines), lamina propria (blank spaces) and muscularis mucosa (black solid lines), are indicated. (*b*) Schematic of mucus layers present in the stomach, small intestine and colon. The mucus layer in the stomach (i) and colon (ii) is composed of a firm inner layer (represented in dark grey) and a loose outer layer (represented in grey). In the small intestine (iii scheme), the mucus is loose and arranged irregularly over the villus. MUC2 is enriched in all structural domains of the intestine, with the exception of the stomach, which is enriched in MUC5A. Relative mucus height (mm) in the stomach, small intestine and colon is shown. The intestinal lumen carries the microbiome and the eukaryome (non-represented). Some pathogens specific to each compartment are shown; these include bacteria (e.g. *Helicobacter*, *Vibrio*, *Escherichia* and *Salmonella*) and parasites (e.g. *Giardia, Entamoeba* and *Cryptosporidium*).

The GI tract contains two anatomical divisions: the upper GI and the lower GI [1]. The upper GI extends from the oral cavity to the small intestine and includes the oesophagus, stomach, duodenum, jejunum and ileum. The lower GI includes the colon, rectum and anus [1]. These anatomical divisions of the GI tract are in addition organized by several layers including the mucosa, the submucosa, the muscularis propria and the connective tissue [2,3]. The mucosal level is home to commensal microbiota, interacts with infectious microbes and comprises subsequent strata: (i) the epithelium facing the intestinal lumen and secreting mucus, (ii) the lamina propria, composed of an elastic connective tissue enriched with collagen, homing fibroblasts, and containing blood vessels, nerves, and immune cells and (iii) the thin muscularis mucosa responsible for the slow intestinal movements supporting intestinal secretion (figure 1*a*).

The epithelial layer is of great interest because it is the physico-chemical and immunological barrier against luminal antigens and enteric pathogens yet allowing the absorption of nutrients and water. For intestinal absorption functions, the epithelium folds into multiple invaginations, making the intestinal surface the largest mucus-covered absorption area in the body (approx. 400 m^2^) [4]. The intestinal epithelium digests and absorbs nutrients by organizing different cells into multiple structures: gastric pits (stomach) and tubular invaginations called crypts (in the colon), which combine with villi (in the small intestine) (figure 1*a*) [4]. The cellular building block of this enteric surface is composed of diverse cells: enterocytes adapted for absorptive metabolic and digestive functions; goblet cells (GC), which secrete mucin, with the newly described specialized GC denominated the ‘sentinel goblet cell’ (senGC) that are localized at the entrances of the colonic crypt and that play a main role in the protection against bacterial infection [5]; enteroendocrine cells, which secrete hormones; Paneth cells, at the bottom of the crypts, which deliver anti-microbial peptides; microfold cells, which are transporters of microbes and particles across the epithelial cell layer, playing a role in mucosal immunity; cup cells of unknown function [6] and chemosensory epithelial cells (Tuft cells), mainly in the small intestine [7]. All of these epithelial cells are derived from the differentiation of the intestinal stem cell (ISC) located at the bottom of the crypts [8]. The intestinal epithelium is renewed every 4–5 days as a result of the proliferation of ISC. Another essential element of the epithelium is the extracellular matrix (ECM), which acts as a scaffold involved in epithelial morphogenesis, differentiation and homeostasis. The ECM is a complex mix of proteins, glycoproteins and glycosaminoglycans secreted by cells, the main functions of which are to provide architectural support to cells. In addition, the ECM is involved in cellular communication by the diffusion of soluble and insoluble molecular components derived from its degradation [9]. In GI tract, the ECM is principally composed of a dense connective network of collagens (collagen type I [70%], type III [20%] and type V [12%]) [10], fibronectin and laminin [11].

The mucus layer is also of prime interest because it forms an interface between the environment of the intestinal lumen and the epithelium, protecting the tissue against microorganisms and toxins (figure 1*b*), shear stress and chemical damage while facilitating colonization by commensal bacteria. Mucus is a two-gel structure (figure 1*b*), there is a firm inner layer, impermeable to bacteria, and its integrity is crucial to avoid direct contact of pathogenic microbes with the epithelium [12,13], and a loose outer layer that interfaces with the microbiota, food products and pathogens [14]. In both layers, a network of glycoprotein containing several mucins secreted by GCs builds the gel; among them, mucin-type 2 (MUC2) is enriched in all structural domains of the intestine, with the exception of the stomach, which is enriched in mucin 5A (MUC5A) (figure 1*b*) [15–17]. In the small intestine, the mucus layer is less defined, and the tips of the villi commonly are not covered by MUC2 (figure 1*b*). In addition, a specific proteome modulated according to tissue specialization along the intestinal tract is present in the mucus [16]. The intestinal microbial ecosystem uses glycans derived from mucins as an energy resource [18–20].

### 1.2. The microbiome as a component of the intestinal tract

According to data obtained from MetaHit and the Human Microbiome Project, the bacterial gut microbiota presents 2172 species classified into 12 different phyla, of which 93.5% belonged to Proteobacteria, Firmicutes, Actinobacteria and Bacteroidetes [21–23]. The specific distribution of these bacteria along the GI tract or, from the lumen to the mucus layer, is determined by specific factors such as pH, oxygen, gradients of antimicrobial peptides (AMP), bile acids and the transit speed [24]. In the case of pH, it is basic in the oesophagus and acidic in the stomach. Then, it gradually returns to basic until the jejunum where it becomes acidic again. From the ileum to the colon, the pH gradually returns to basic (6–7.5 in the small intestine) and is close to 7 in the colon. The drop in pH at the ileocecal junction is attributed to the production of short-chain fatty acids by bacterial fermentation. There is also an oxygen gradient toward the GI with high levels in the upper GI and lower levels of oxygen in the distal GI [25]. Breathing air at sea level has an oxygen pressure (PO_2_) of approximately 145 mmHg (approx. 21% O_2_). By contrast, in the lumen of a healthy colon, there is a PO_2_ of less than 10 mmHg. PO_2_ levels decrease along the radial axis of the intestinal submucosa to the lumen. The amounts of mucus are another element that determines the composition of the microbiome (figure 1*b*) [26]. These physiological parameters regulate the total number of microbes per gram of faeces (from the stomach with 10^2^ to the distal colon with 10^12^ microbes) [25] and influence the variability of the microbiome. In the upper GI, there are mainly aerobes or facultative anaerobic microorganisms that are able to live in an acidic and oxygenated environment (e.g. *Helicobacter*, *Vibrio*, *Giardia*, *Lactobacillus*, *Streptococcus* etc.) and in the lower GI, the microbe population is made up of facultative anaerobes and strict anaerobes organisms that grown in basic–neutral pH media little or no oxygen flow (e.g. *Escherichia*, *Shigella*, *Salmonella*, *Listeria*, *Cryptosporidium*, *Blastocystis*, *Entamoeba* etc.) (figure 1*b*).

All these commensal organisms play important roles in intestinal homeostasis such as protection against pathogens [27], helping to maintain the integrity of the mucosal barrier [28], providing nutritional elements such as vitamins [29] and aiding the mucosal intestinal immune response [27,28,30]. In addition, the bacterial microbiota is essential for ensuring important metabolic functions that cannot be performed by the host. For instance, some obligate anaerobic bacteria are able to degrade complex non-digestible carbohydrates (fibbres) to produce short-chain fatty acids such as acetate, butyrate and propionate [31]. These lipids, rather than glucose, are the preferred energy substrate for colon epithelial cells. In addition, the increase of plant-derived fatty acids in diets leads to changes in intestinal morphology characterized by elongated villus structures with an increased number of epithelial cells and a reduced rate of epithelial proliferation [32]. In turn, the fat-rich diet changes the composition of the bacterial microbiota [32], suggesting the need to maintain a balance among tissue renewal, bacteria and the immune system in order to ensure intestine homeostasis [28]. Dysbiosis, originating from a disorder of the gut microbiota caused by excessively fatty diets or antimicrobial drugs, is involved in intestinal diseases (e.g. IBD, and metabolic diseases such as obesity and diabetes).

The impact of other microbes on intestinal homeostasis is poorly studied, despite the fact that several eukaryotic microbes (the eukaryome) are common residents of the healthy human intestine. For example, the influence of intestinal colonization by a protozoan on the composition of the bacterial microbiota has been demonstrated by the presence of *Blastocystis* [33,34], a component of the eukaryome detected with high prevalence as a commensal in healthy people. In addition, *Blastocystis* has also been found in association with other protozoa such as non-pathogenic *Entamoeba* species [35]. Co-colonization by *Entamoeba* spp. and *Blastocystis* *hominis* exists in healthy human populations [36]. Some recent data indicate that the protozoan *Tritrichomonas musculis* is a commensal of the intestine [37] and protects mice from bacterial infections. Over 140 fungal genera have been discovered as permanent or transient biota in the intestinal tract; many are either beneficial or commensal (e.g. *Saccharomyces*), and are considered probiotics with a role in the treatment of diarrhoea [38]. Finally, the virome comprises all the virus-like particles associated with the GI tract; it is mainly composed of two groups, eukaryotic viruses and prokaryotic virus also known as bacteriophages [39]. Eukaryotic viruses are able to interact with human body as pathogens, agents causing acute or persistent infections, or as benign agents, or even symbiotic agents without affecting the human body [40]. Bacteriophages are the principal component of the GI tract virome by their ability to infect bacteria and archaea [41,42]. Their distribution across the GI tract is varied but average is 109 particles per gram [39]. Bacteriophages from the human gut principally belong to the virus families Myoviridae, Podoviridae and Siphoviridae [43] and in general, opposed to bacterial population, the bacteriophage community in the human gut is highly personalized [44].

All these findings suggest a specific role that can be expected from each intestinal microbiome constituent to govern the balance of the intestinal ecosystem, a hypothesis constituting the subject of emerging research.

### 1.3. Microbiota and intestine changes during intestinal infection

Microbial pathogens such as parasites, bacteria and viruses, that are normally only present transiently in the intestine, can colonize or invade intestinal tissue under abnormal conditions [45]. In humans, the most common intestinal parasitic infections are caused by helminths and protozoans. Intestinal helminths are worms, of which the most common cases are nematodes, cestodes and trematodes [46,47], represented by the worldwide disseminated species: *Ascaris lumbricoides*, *Trichuris trichiuria*, *Ancylostoma duodenale* and *Necator americanicus* [46]. These parasites infect 1 billion people worldwide, causing significant ill health [48]. Likewise, the most common intestinal protozoan parasites in humans are *Giardia intestinalis*, *Entamoeba* spp., *Cyclospora* and *Cryptosporidium* spp. [47]. The presence of intestinal protists such as *Entamoeba*, *G. intestinalis* and *B. hominus*, induces significant changes in the bacterial diversity of the microbiome, and consequently in intestinal homeostasis [49]. Among the pathogenic bacteria invading the human intestine, the most common groups are *Helicobacter pylori*, *Vibrio cholerae*, *Campylobacter*, *Salmonella* spp., *Listeria monocytogenes*, *Shigella* spp. and virulent strains of *Escherichia coli* [50], while the main target of invasive bacteria are the cells lining the epithelium. In the case of virus, rotaviruses are the major cause of gastroenteritis in infants and young children [51]. Likewise, Norwalk and Norwalk-like viruses, are the major cause of gastroenteritis in adults and older children [52]. Noroviruses are also the other principal aetiological agents of viral gastroenteritis [53].

The features above indicate the great cellular complexity present in the intestine. To be able to understand human GI physiology under healthy and pathological conditions, it is necessary to determine the role of cellular activities, as well as the biochemical and mechanical signals involved therein. For this reason, for many years, researchers have been developing *in vitro*/*ex vivo* systems that could mimic the cellular diversity present in the human intestine, the flow of metabolites and peristaltic movements. In this review, we summarized data obtained with simple and complex systems used to assess the interaction between human intestine and commensal or pathogenic microbes; their advantages and limitations are also discussed.

## 2. Experimental models established for the study of intestinal infections and beyond

### 2.1. Monolayers of epithelial cells

The use of animals as a model to understand human diseases is a common approach [54]. However, the applicability and validity of animals to study interactions between intestine and microbes is permanently questioned mainly because in the case of obligated pathogens, animals do not reflect the tissue and immune responses observed in humans. To overcome these weaknesses, there is growing interest in the development of *in vitro* models capable of reproducing human intestinal function. Among the systems developed are included the culture of cells, which uses cells removed from tissues and placed in multiwell microplates or Petri dishes as culture vessels. In the correct environment, those cells grown in a monolayer (two-dimensional (2D) conditions) and are used to evaluate cellular responses to a specific stimulus. These *in vitro* cultures of epithelial cells have for many years been the most used system to study the interaction of pathogenic microbes and the intestine and important discoveries and advances in our understanding of infectious disease. Their principal advantages are the cells' ability to polarize and form cell–cell tight junctions, promoting the integrity of the epithelial barrier, they are reproducible, and many molecular and biochemical tools can be implemented in them [55]. As major disadvantages, cells grow in 2D *in vitro* conditions, limiting the expression of tissue-specific factors, such as mucus, and in general, they are used to study the interaction between a single microbe and a single or a few types of host cell. The monolayers are derived from the primary cell or from intestinal tumours. Primary cells in culture are able to proliferate *in vitro* without, or with very few, subculture steps, retaining some *in vivo* functionality [56]. Interesting data have been obtained with primary gastric cells interacting with *Helicobacter pylori*, thereby confirming that this bacterium attaches to cell–cell interfaces [57]. Two non-tumour intestinal cell lines with epithelial phenotypes (FHC CRL-1831 and CCD 841 CoN) have been, respectively, used to evaluate the effective antimicrobial activity of nanocomposites against *Escherichia coli* and *Staphylococcus aureus* [58], or to demonstrate the positive effect of several probiotics to decrease the adhesion of several *Clostridium difficile* strains [59]. Likewise, the low cytotoxicity effect of 19 new fluoro-benzimidazole derivatives was demonstrated in cell line CCD 841 CoN, in contrast to the high inhibitory activity of those molecules against *Escherichia coli* O157:H7, *Escherichia coli* ATCC 8739 *Escherichia coli* ATCC 35218 and *Salmonella typhimurium* ATCC 1331 [60].

The culture of tumour cells is a much more popular system to study cellular physiology during infection. As an advantage, these cell cultures are relatively inexpensive, the cells survive after several subculture steps, and there are many options for applying technologies of genetic manipulation. However, the use of cancer cell lines is limited in terms of establishing the functional character of human intestine, in that they derive from a sole cell linage [61]. The most popular human colon tumorigenic cell lines employed *in vitro* are Caco-2 and HT-29, derived from a colon tumour and able to differentiate, respectively, into enterocyte-like and mucus-producing GC. These cells have been widely used under monoculture or co-culture conditions [19,62–64]. Extensive research has been performed using these cells to investigate the cellular and molecular mechanisms by which pathogenic microbes (parasites, bacteria or virus) create structural lesions on human tissues [65–67]. These will not be further discussed in this review and have been well summarized previously [3,68–70].

In the following sections are described three-dimensional (3D) models considered as devices that reconstruct the 3D architecture of human intestine, are able to recapitulate the most basic functional units present in the original tissue, express the functions of differentiate intestinal epithelium (e.g. mucus production, villi) and respond to chemical, biological and physical interventions.

### 2.2. Human intestinal explants

**Figure 2.**
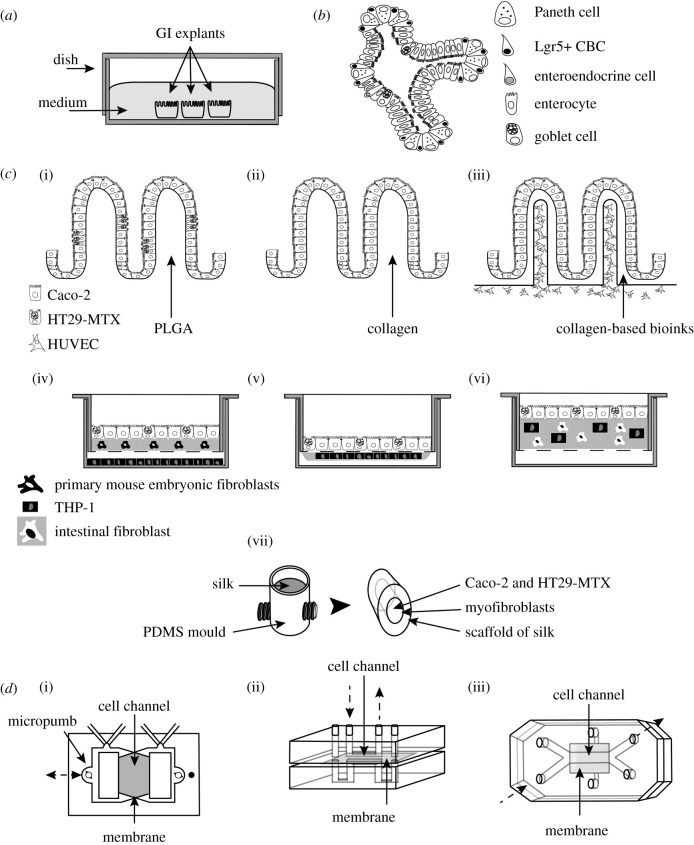
Schematic of major fate of the diverse intestinal models discussed in this review. (*a*) Tissue explants. (*b*) Organoids: the villus-like domains, crypt-like domains and epithelial cell lineages present in organoids are shown. (*c*) Scaffold-based 3D models. (i), PLGA scaffold with villous features containing Caco-2 and HT29-MTX cells. (ii) Scaffold with villous features made with collagen, seeded with Caco-2 cells. (iii) Collagen-based scaffold with villous features in which have been incorporated a blood capillary structure. It contains Caco-2 and HUVEC cells. (iv) 3D-trans-well model made with collagen as the scaffold. In the apical side of the collagen, were seeded Caco-2 and HT29-MTX cells. Within collagen, there were primary mouse embryonic fibroblasts. In the base of the well, were added THP-1 cells differentiated to macrophages. (v) 3D-trans-well model made with collagen as the scaffold. Over the collagen were added Caco-2 and HT29-MTX cells. On the opposite side of the membrane, a drop of collagen-containing THP-1 macrophages were seeded. (vi) 3D-trans-well model made with collagen as the scaffold. Caco-2 and HT29-MTX cells were seeded over the collagen as the epithelial layer. To the lamina propria-like layer were added intestinal fibroblast and THP-1 macrophages. (vii) Scaffold made with silk fibroin as the substratum obtained from a model of PDMS. Human intestinal myofibroblasts were seeded in the external side of the model to reproduce the lamina propria. Caco-2 and HT29-MTX cells were added in the hollow channel of the scaffold as the epithelial layer. (*d*) Organ-on-a-chip models. (i) Intestinal chip made with PDMS. This device has two independent channels separated by a membrane. Caco-2 cells were inoculated on the upper side of the membrane previously coated with collagen. (ii) Chip made with two PDMS sheets, separated by a collagen-coated permeable membrane. Two vertical microchannels connect the upper (apical side) and lower side (basal side) of the membrane. Caco-2 cells were cultured on the apical side. (iii) ‘Gut-on-a-chip' model. It has two microfluidic channels separated by a porous flexible membrane coated with extracellular matrix proteins. Microfluidic flux is represented with dotted arrows.

Experimentation with intestinal explants has rendered possible the study of tissue interactions with microbiota, bacteria or parasites in a 3D context. This is a technology devoted to maintaining a whole organ, or portions of an organ, in culture by the use of specialized media, substrates and atmospheres (figure 2*a*) [71]. Although their major disadvantages are the difficulty of obtaining tissue samples and the short explant viability, these models are very attractive for studying the interaction among human tissues and microbiota. For instance, this technology has been employed in several studies: to analyse the interaction of the intestinal microbiome; to detect the presence and effect of different microorganism populations [30,72,73]; to determine that bacterial outer membrane vesicles are internalized by polarized cells and it is an effective strategy to communicate signals among beneficial gut bacteria and to modulate host responses [74]; and to observe in ileum explants that alterations in the transportome system in Crohn's disease are partially restored by commensal bacteria [75]. The human colon explant model reproduced early pathogenic events following *Shigella flexneri* infection and the mechanisms involved [76]. Furthermore, loading *Entamoeba histolytica* on human colon explants demonstrated that amoebic cysteine proteinase A5 promotes the activation of human matrix metalloproteinase-3, which in turn activates human matrix metalloproteinase-1, leading to remodelling of the fibrillar collagen and mucosa invasion [77]. Likewise, transcriptome profiling of amoebic gene expression shows the potential role of carbohydrate metabolism in colon invasion by *E. histolytica* [78], and it was determined that the rapid changes in gene expression profiles, rather than genetic derivation, account for the invasive phenotype of a single virulent amoebic isolate [79]. The infection of ileum explants with *Cryptosporidium parvum* or *Cryptosporidium hominis* causes, in the early stages, the overexpression of osteoprotegerin, which prevents the early apoptosis of human cells. The system also allows the parasite to achieve its complete life cycle, a feature not obtained with cell cultures [80]. Human intestinal explants have also been used to study immunological response. For example, the administration of the Cholera toxin B subunit reduces mucosal Th1 cell signalling [81]. Although impressive data have been obtained with human explants, as was mentioned, the major disadvantages of this model lie in the difficulty of obtaining human tissue and the short duration of viability of the explant.

### 2.3. Organoid models

Although at present the term intestinal organoids continues to be used in a broad and unspecific manner, herein referred to as a 3D miniaturized intestine able to grow *in vitro* and that is characterized by its ability to generate villus-like structures and crypt-like proliferative zones (figure 2*b*) [82,83]. Organoids derive from the proliferation of ISC, which are isolated from embryonic or mature crypts. Under specific cell-culture conditions, ISC proliferate and form spheres (from 100 µm to 700 µm) and, promoting their differentiation into intestinal epithelium, mesenchyme and lumen-like structures [84]. The most widely used method to develop organoids was described in 2009. This methodology isolates intestinal crypts further cultured in collagen matrix leading to the subsequent development of villus-like and crypt-like structures [85]. Later works have improved the method to allow the differentiation of intestinal organoids into enterocyte, goblet cell, Paneth cell and enteroendocrine lineages (figure 2*b*) [86]. Likewise, according to the ISC sources, organoids could be referred to as ‘enteroid' when the cells come from the small intestine, and ‘colonoid' when cells derive from colon [87]. The advantage of organoids includes the presence of 3D structures with villus-like domains able to maintain cellular polarization toward the tissue. They also possess crypt-like domains with proliferative regions that contain cells expressing ISC markers and that are able to differentiate into all of the epithelial cell lineages (figure 2*b*) [69]. Furthermore, intestinal organoids can be genetically manipulated and, if they were obtained from embryonic stem cells, they can be expanded indefinitely and cryopreserved, permitting long-term storage [88]. However, as these models represent only the general aspects of the epithelial layer, their use presents some limitations, including the lack of immune, nerve or vascular cells. Another important point to consider is organoid morphology as a closed system. As they have a spherical shape, the intestinal lumen occurs inside the device. To overcome this limitation, the uses of organoids require modification of certain technical approaches, such as mechanical shearing to promote the solubilization of the semi-solid spherical structure and further, to generate a polarized epithelial layer in trans-well chambers, or the microinjection of compounds to avoid the barrier effect [89,90]. For instance, a high-throughput organoid microinjection system was recently developed that allowed for the correct delivery of samples into the organoid's lumen [91]. In organoids deriving from duodenal biopsies from gluten-intolerant patients, the barrier function was improved after the microinjection of butyrate, lactate and polysaccharide A [92]. All of these aspects unquestionably situate organoids as excellent models for studying the interaction between the intestinal epithelium and microbes. For example, the co-culture of mouse intestinal organoids and human lamina propria lymphocytes in the presence of *Lactobacillus reuteri* D8, was able to repair the epithelial damage caused by TNF-α treatment, improving intestinal barrier function and epithelial layer proliferation [93]. In organoids derived from human pluripotent ISC localized near the immature epithelium, the interaction between cells and the non-pathogenic strain of *Escherichia coli* results in stable host–microbe symbiosis, permitting the maturation of both the mucus layer and the epithelial barrier [94]. Human colonoids were used to generate a differentiated monolayer in trans-wells inserts. In them, infection with enterohaemorrhagic *Escherichia coli* (EHEC) showed efficient bacterial attachment to the apical surface of mucus-producing cell monolayers, affecting brush border integrity [95]. Furthermore, with organoids, it has been possible to overcome the obstacle that the anaerobic requirements represent of many organisms. For instance, deposition by microinjection of the obligate anaerobe bacterium *Clostridium difficile* into the lumen of organoids, reveals disruption of organoid's epithelium, loss of paracellular barrier function, loss of polarity, and finally, reduction of mucus production [96,97]. Another study demonstrated that the pathological mechanism of *Clostridium difficile* depends on the expression of the Frizzled receptor [98]. Similarly, microinjection of the non-pathogenic commensal strain of *Escherichia coli* SGUC183 or of the clinical isolate *E. coli* (PT29S) O157:H7 into the organoid's lumen exhibits bacterial viability after 20 h. The commensal bacterial strain does not cause damage in the lumen; by contrast, a clinical virulent isolate producing the Shiga toxin gives rise to cellular damage, activation of the SOS stress response by production of reactive oxygen species and the upregulation of inflammatory response genes, including interleukin 8 [99]. Microinjection of *Salmonella enterica* serovar *Typhimurium* promotes large-scale transcriptional changes in organoids, including in genes linked to the proinflammatory response [100]. Microinjection of *Helicobacter pylori* into the lumen of the organoids demonstrates bacterial adhesion to the epithelium leading to the disruption of apical cell–cell junctions, supporting the idea that organoids reflect the pathology in the original tissue [101–103]. The infection with *Shigella flexneri* of epithelial layer derived from enteroids shows basolateral invasion and disruption of tight junctions. In this system were observed bacterial replication, actin tails and increase in proinflammatory signals [104]. Likewise, the infection process of *S. flexneri* was evaluated in disaggregated organoids from human enteroids [105]. In this system was observed how the apical invasion by *S. flexneri* was increased (10-fold) when the enteroids were differentiated to M cells. Also, invasion increased when the parasite infection was via the basolateral surface. Finally, the secretion of interleukin-8 and MUC2 were also increased after *S. flexneri* infection [105]. Similarly, the study of *Escherichia coli* infection process was studied in polarized organoids. Those 3D models were disaggregated and grown on trans-well inserts and infected with pathogenic strains showing novel adherence phenotypes [106]. Impressive data showed that microinjection of *Cryptosporidium* spp. [107,108] were able (as with tissue explants) to infect human enteroids with completion of the parasite's life cycle following dynamic regulation of transcripts related to the life cycle [107]. These findings have opened great avenues for further experimentations with *Cryptosporidium*, which is unable to proliferate *in vitro*. Recently a new model was developed, derived from stem-cell-derived spheroids seeded and cultivated on trans-well insert by an ‘air–liquid interface' [109]. These culture conditions allow the infection with *C. parvum*, and the completion of the parasite life cycle and parasite expansion to generate viable oocysts.

Viral gastroenteritis is a major cause of morbidity and mortality worldwide. Our understanding of the pathogenesis of human intestinal viral infection is limited and comes primarily from clinical investigation of the infection. The use of human organoids has provided new data on this important health problem [110]. For example, organoids derived from patient samples have been developed as a model for understanding the process of rotavirus infection [111–113]. The experimental setting recapitulates the essential characteristics of the *in vivo* tissue architecture allowing viral infection and water influx to the intestinal lumen [113]. The system demonstrated the great importance of the PI3 K-Akt-mTOR signalling pathway and the exchange of GDP/GTP in the small GTPase protein Rac1 on rotavirus infection [114,115]. As mentioned earlier, noroviruses are another major cause of gastroenteritis in humans. Recently, there has been successful adaptation of the use of a human intestinal epithelial monolayer derived from organoids as a norovirus culture system [116–118]. In addition, although infection with SARS-CoV-2 virus typically presents respiratory disease, emerging clinical reports indicate possible viral replication in the intestinal epithelium. In this case, through the use of intestinal organoids, viral infection of human enteric cells has been determined associated with infection of respiratory cells [119,120].

In conclusion, organoids reiterate anatomical aspects of the human intestine such as villus-like crypt structures. This tissue model is generalized to explore the interaction of human intestine and commensal, pathogenic bacteria or virus. Although their use as models to study the interaction between the parasite and intestinal tissue will certainly require overcoming various technical problems, it is undoubtedly a powerful tool for understanding the process and signalling pathways evoked during intestinal infections.

### 2.4. Scaffold-based three-dimensional models

Another revolutionary type of 3D intestinal model are scaffold-based 3D models. Tissue engineering has provided specialized biomaterials as substrates for the deposition of the ECM and cells to mimic the architecture of tissue. These biomaterials require biocompatibility in order to allow for cell adhesion, porosity to ensure adequate diffusion of nutrients, and the capability to act as guides for the organization of cells, leading to the reproduction of their general layout in the human gut. Two types of materials are regularly used: synthetic materials (e.g. polydimethylsiloxane (PDMS), polyglycolic acid (PGA) and poly-lactic-co-glycolic acid (PLGA)) and natural extracellular materials (e.g. collagen or matrigel) [121]. PDMS is the most popular of these materials due to its low cost and relatively easy handling, as well as its high permeability to gases such as oxygen [122]. Although cell scaffolds reproduce, in many structural and functional aspects, the original tissue, they entail some limitations, such as incomplete 3D tissue architecture, reduced multicellular complexity, and the absence of physical forces demonstrated by natural tissues [70]. Nevertheless, scaffold-based 3D models have generated important results.

Scaffold-based 3D models build with synthetic materials such as PLGA with Caco-2 and/or HT29-MTX cells recapitulate cell differentiation along the villus axis and mucus (figure 2*c*(i)) [123,124]. Following the interaction of intestinal cells with the microbiota, it was successfully proven that two probiotics (*Lactobacillus gasseri* and *E. coli* Nissle 1917) were able to prevent adhesion and invasion of the pathogens *Salmonella typhimurium* 14038 and *Pseudomonas aeruginosa* 15692 [125]. Employing the sacrificial mould procedure (PDMS villi mould–alginate reverse mould–3D hydrogel mould), a 3D villi scaffold was built with collagen as the substrate and Caco-2 cells (figure 2*c*(ii)) [126]. This procedure permits the easier separation of the hydrogel structure and creates a scaffold with better-conserved detail. The gene expression profile of differentiated cells significantly changed in comparison to cells in 2D culture; mainly, there is a significant increase in mucin-gene family expression. *Salmonella typhimurium* was able to invade the crypt part of the 3D scaffold, while no invasion was observed in cells on villi tips [126]. Recently, by means of the cell-printing process, a blood capillary structure was incorporated into the villi (figure 2*c*(iii)) [127]. This system revealed a higher rate of cell growth, overexpression of MUC17, and enhancement of barrier function. Another interesting scaffold was constructed by means of the combination of PDMS and silk fibroin as substratum, hosting human intestinal myofibroblasts, Caco-2 and HT29-MTX cells (figure 2*c*(vii)) [128]. Mucus accumulates on the epithelial surface, low oxygen tension was observed in the lumen and bacteria colonize the scaffold (e.g. *Yersinia* or probiotic *Lactobacillus rhamnosus* GG) [128]. The same system reproduced a long-term infection by *Cryptosporidium parvum*. The parasite developed asexual and sexual stages or was able to form new oocytes. Also, ablation, blunting, microvilli distortion in infected epithelial cells were observed [129]. Recently, the use of this device, was extended with the co-culture of human intestinal myofibroblasts and cells from human intestinal enteroids and the infection with *C. parvum* [130].

Scaffold-based 3D models built with natural extracellular matrices open emerging alternatives for the study of intestinal infections, for example, the very recent approach that the use of Caco-2 cells reseeded over decellularized porcine jejunal segments used as supporting material [131]. The hybrid human–porcine 3D model revealed intestinal features such as epithelial polarization and recapitulated the pathological process of *Campylobacter jejuni* previously observed in animal models; it particularly confirmed the role in the infection of the small regulatory RNA pair CJnc180/190 [132]. There are complex 3D scaffolds that employ purified collagen as support; in these, different types of cell lines, including immune cells, have been embedded, resulting in enhancement of physiological relevance. For example, for the evaluation of drug absorption, the 3D scaffold was built with Caco-2 and HT29-MTX cells seeded over a collagen matrix, primary mouse embryonic fibroblasts were found within the collagen, and human monocytes THP-1 differentiated into macrophages were seeded into the base of the well (figure 2*c*(iv)) [133]. In this *cells–collagen* *+* *cell–filter–cell* scaffold the following was observed: mucus production, overexpression of the efflux transporter *BCRP* gene, and significant values of absorption for specific drugs. Other scaffolds employed the compartmentalization properties of trans-well chambers: the mix of Caco-2 and HT29-MTX cells was seeded on the apical side of the filter and on the opposite side, a drop of collagen-containing THP-1 macrophages was deposited (figure 2*c*(v)). This *cell–filter-collagen* *+* *cells* scaffold reproduced the features of the small intestine microbiome after the addition of a set of eight commensal bacterial strains (*Enterococcus faecalis*, LMG-7937; *Escherichia coli*, DSM-18039; *Streptococcus salivarius*, LMG-11489; *Streptococcus mitis*, LMG-14557; *Lactobacillus plantarum*, LMG 1284; *Veillonella parvula*, DSM-2008; *Veillonella atypica*, DSM-20739 and *Prevotella intermedia*, DSM-20706) [134]. Another complex scaffold composed of decellularized porcine small intestinal submucosal collagen, Caco-2 cells, and primary human microvascular endothelial cells supplemented with peripheral blood leucocytes providing the vascular immune system was infected with *Salmonella*; it showed the communication of epithelial, endothelial, monocytes and natural killer cells among each other and with the pathogen [135]. A recently built 3D scaffold contains Caco-2/TC7 and HT29-MTX cells seeded on top of a collagen layer containing CCD-18Co human fibroblasts and THP-1 differentiated macrophages to mimic the basal lamina propria (figure 2*c*(vi)). This *cells–collagen* *+* *cells* scaffold presents intestinal features such as mucus production, epithelial cell polarization, fibroblast networking and cytokine production. The combination of imaging, omics and the evaluation of immune responses led to the analysis of the earlier steps of infection evoked by *Entamoeba histolytica* [136]. The data highlighted several virulence markers previously reported in the explants model or in patients with intestinal amoebiasis; in addition, new regulatory factors in the amoebic invasive process, including non-coding RNAs, ion transporters and nuclear receptors, were identified. For the first time, it was also observed that *Entamoeba histolytica* swallowed pieces of mucus after detaching them from the epithelium, and that immune and anti-microbial human defences are inhibited when the parasite interacts with the cells [136].

In conclusion, scaffold-based 3D models recapitulate diverse cell types presents in the intestinal epithelium. The advantages are the following: (i) versatility, in that all of its components can be varied independently, given the opportunity of adding or removing elements according to the study objectives within a 3D context, and (ii) the possibility of combining 3D scaffolds and cells derived from human intestinal explants or organoids. Tissue scaffold models reproduce architecture of living tissues, promoting the correct intestinal cell differentiation and facilitating the study of the earlier steps of pathogenic processes in conditions similar to the original tissue.

### 2.5. Organ-on-a-chip models

Microengineering approaches (e.g. soft lithography, moulding and micromachining) have developed 3D devices known as ‘organs-on-a-chip' [137]. These chips are models that control the environment around cells thanks to their micropatterned surfaces, which support the correct spatial arrangement of the cells and help to control the gradients of the biomolecules by microfluidic applications. Organ-on-a-chip systems demonstrate many advantages, including a 3D environment that emulates tissue structure with the presence of cell lines or stem cells, and microenvironmental cues with microfluidic systems that permit the mimicking of the cellular microenvironment [138]. Their principal weakness lies in the implementation of highly complex structures requiring qualified personnel for their design, and PDMS, the material most commonly used for chip construction, which absorbs small hydrophobic molecules that could interfere with some drug-screening studies [70]. One of the first intestinal chips was developed in 2008 (figure 2*d*(i)) [139]. This device made with PDMS is divided into two independent microfabricated hollow channels separated by a polyester semipermeable membrane. To support the inoculation and culture of Caco-2 cells, the upper side of the membrane was coated with collagen. The cells showed polarization and were successfully maintained for 30 days. An additional system was developed to evaluate intestinal absorption (figure 2*d*(ii)) [140]; this system was composed of two PDMS sheets, separated by a collagen-coated permeable membrane. Two vertical microchannels connect the upper (apical side) and lower side (basal side) of the membrane. Caco-2 cells were cultured on the apical side. Those conditions were able to support the precise control of the fluid, mimicking vascular flow. Although these models were an important step in emulating general intestinal functions, they were not able to reproduce physiological characteristics such as villi structure, the presence of multiple specialized cells and peristaltic movements, all necessary to simulate the symbiotic relationship between intestinal cells and resident bacteria [141]. To overcome these limitations, in 2012, the same microengineering principles employed in the ‘lung-on-a-chip', were used to build the ‘gut-on-a-chip' (figure 2*d*(iii)) [142]. This chip incorporates two microfluidic channels separately by means of a porous flexible membrane coated with ECM proteins. In the upper side, containing Caco-2 cells, the cell polarizes and forms villi-like structure by their exposition to micro-flow and cyclic strain to mimic peristaltic motion. The chip showed successful interaction, for one week, between human cells and the commensal bacterium *Lactobacillus rhamnosus* GG. The intestinal villi-like structure differentiates, produces mucus, and increases in the activity of the drug-metabolizing cytochrome P450 3A4 (CYP3A4) enzyme, both important during the morphogenesis of villi-like structures [143]. In this model, it was also demonstrated that the infection of *Shigella flexneri* is significantly increased by the villi-like structure [144]. To engineer the intestinal tissue–tissue interface, on the lower side of the membrane were added microvascular endothelial cells [145]. Under these conditions, the interaction was studied between cell and commensal microbes (*Lactobacillus acidophilus*, *Lactobacillus plantarum, Lactobacillus paracasei*, *Bifidobacterium breve*, *Bifidobacterium longum* and *Bifidobacterium infantis*). Bacterial contact modified the gene expression of Caco-2 cells, rendering it similar to the gene expression of normal human ileum. It was also demonstrated that peristaltic motion and the administration of therapeutic probiotic formulation avoided the damage in the intestinal barrier promoted by pathogenic strains of *Escherichia coli* and protected against bacterial overgrowth [145]. Another step forward taken to improve the gut-on-a-chip models was their combination with enteroids cultured from duodenal biopsies [146], or lines from induced pluripotent stem cells (iPSC), which were fragmented and seeded on the apical side of the ECM-coated membrane with fluid-flow and peristalsis-like deformations to the basal side [147]. Likewise, enteroid cells were able to produce villus-like structures with multilineage differentiation and, as expected, transcriptomic analysis revealed a host-defence response to infection of the human duodenum [146]. Recently, also was demonstrated that EHEC led to greater epithelial injury when exposed to metabolites derived from the human gut microbiome (4-methyl benzoic acid, 3,4-dimethylbenzoic acid, hexanoic acid and heptanoic acid). The authors suggest that these metabolites could be the reason why some human populations are more susceptible to EHEC infection [148]. To study the interaction of aerobic and anaerobic human gut microbiota and intestinal cells, a microscale oxygen sensor was incorporated to measure the amount of oxygen *in situ* [149]. Intestinal epithelium and microvascular endothelium were grown in parallel chambers, separated by an ECM-coated porous membrane. Then, the device was placed within an anaerobic chamber to establish oxygen gradients between the upper and lower chamber, establishing differential amounts of oxygen between the epithelial cells and endothelial cells. The set-up permits the stable co-culture of highly complex communities of anaerobic and aerobic commensal bacteria and the intestinal epithelium in the same channel [149].

The HuMiX microfluidic device was developed to study the GI human—microbe interface [150,151]. This is a human–microbial crosstalk system composed of three microfluidic chambers separated by semipermeable membranes. The lower chamber is a perfusion chamber, the middle chamber is the cell-culture chamber and the upper chamber is the microbial-culture chamber [151]. The system can be used to grow human cell or bacterial strains under oxygen and biomolecule gradients [150]. In this system, a successful co-culture either with a facultative anaerobe bacterial strain, *Lactobacillus rhamnosus* GG, or in combination with the obligate anaerobe bacterial strains, *Bacteroides caccae*, was obtained [150]. Organ-on-a-chip technology has been employed also to develop an *in vitro* model of the human colon [152]. Colonoids were obtained from re-sections or endoscopic tissue biopsies; these were disaggregated, and the cells were seeded in the chip above described. Colon cells were able to produce a polarized epithelium containing GC carrying mucus granules and importantly, a bilayer structure of mucus over the epithelium. All of these properties render this colon chip a new tool for the analysis of the role of mucus during commensal or pathogenic infections. Finally, it is important to highlight that, in all of these organ-on-a-chip models, the continuous fluid flow removes secreted molecules that might suppress villi formation, emphasizing the importance of this aspect in the design of the chip models [153–155].

The complexity of microfluidic chips has gained popularity. A recent study combines a microfluidic chip and a 3D villi scaffold to culture Caco-2 cells [156]. This system allows the formation of an epithelial barrier across the membrane or villi surface and high activity of the cytochrome P450 3A4. Another example combines a micrometre resolution membrane that is synthesized from rat-tail type-I collagen in a microfluidic device with apical and basolateral chambers [157]. However, to date, neither of these two systems has been employed to explore intestinal interaction with commensal bacteria, virus, pathogens or parasites.

In conclusion, despite the fact that organ-on-a-chip technologies will undoubtedly be required to overcome methodological limitations, their use will aid in understanding the role of important factors (e.g. tissue mechanics, microbiota, daily diet) on microbial infectious processes. The combination of organoids with microfluidics has opened alternatives for studying the interaction between human gut and microbes, taking into account oxygen rates and peristaltic movements.

## 3. Conclusion

Fundamental research on infectious diseases affecting humans has been limited in the past by the lack of robust experimental models that reproduce pathogen–host interactions in an environment relevant to the disease. This fact motivated the development of diverse 3D intestinal models, their application offers an excellent opportunity to experiment with devices closer to the native intestine. The establishment of organoids partly solves the technological limitations that the tissue explants present (e.g. scarce disposition of human tissues and their short life span). Organoids summarize many architectural aspects of the intestine and have been mainly used to explore organ interaction with commensal or pathogenic bacteria and viruses, while few cases have used organoids to study the pathological stages triggered by intestinal parasites. This model could rapidly spread in parasitology studies due to the validation of organoid disaggregation to form epithelial layers. Parasite–intestine interaction tests, in the early stages of infection, can be clearly enriched by the implementation of scaffold-based 3D models due to their nature of open systems. In addition, organ-on-a-chip technologies are able to combine cells or organoids with microfluidics, opening opportunities for complexes environments to examine intestinal invasion by for parasites (table 1).

The field of tissue engineering has also enriched multidisciplinary research in order to resolve the limitations that these 3D models present, in addition the teams, composed of biologists, engineers, physicists, mathematicians and clinicians, have been working together to design and develop more realistic 3D intestinal models. Behind their complex engineering, experimentation with these models actually poses great technological challenges. For example, in cell biology, the acquisition of microscope images (in particular, live imaging) requires instruments with greater penetration depth and multiple wavelengths, faster acquisition, and reduced phototoxicity. All of these parameters are only found combined in a few microscopes [158]. Another important challenge comprises the analysis of microscope images requiring mathematical tools and computing that produce large sets of volumetric data; indeed, reducing the computational load is an important task that will complement image analysis with all-omics approaches.

In that a unique model cannot afford the entire picture of humans, we think that, only when employed in conjunction, the data obtained with each of the different 3D models will increase our knowledge concerning the processes activated during the interaction of bacteria or parasites and the human intestine.

## Data accessibility

This article does not contain any additional data.
